# Expression of cell cycle proteins in male breast carcinoma

**DOI:** 10.1186/1477-7819-8-10

**Published:** 2010-02-12

**Authors:** Rani Kanthan, Isabella Fried, Theresa Rueckl, Jenna-Lynn Senger, Selliah Chandra Kanthan

**Affiliations:** 1 Department of Pathology and Laboratory Sciences, Royal University Hospital, Saskatoon, SK, Canada; 2 Department of Dermatology, University of Medicine, Graz Austria; 3 Department of Surgery, Royal University Hospital, Saskatoon, SK, Canada

## Abstract

**Introduction:**

Male breast cancer (MBC) is a rare, yet potentially aggressive disease. Although literature regarding female breast cancer (FBC) is extensive, little is known about the etiopathogenesis of male breast cancer. Studies from our laboratory show that MBCs have a distinct immunophenotypic profile, suggesting that the etiopathogenesis of MBC is different from FBCs. The aim of this study was to evaluate and correlate the immunohistochemical expression of cell cycle proteins in male breast carcinoma to significant clinico-biological endpoints.

**Methods:**

75 cases of MBC were identified using the records of the Saskatchewan Cancer Agency over 26 years (1970-1996). Cases were reviewed and analyzed for the immunohistochemical expression of PCNA, Ki67, p27, p16, p57, p21, cyclin-D1 and c-myc and correlated to clinico-biological endpoints of tumor size, node status, stage of the disease, and disease free survival (DFS).

**Results:**

Decreased DFS was observed in the majority of tumors that overexpressed PCNA (98%, p = 0.004). The overexpression of PCNA was inversely correlated to the expression of Ki67 which was predominantly negative (78.3%). Cyclin D1 was overexpressed in 83.7% of cases. Cyclin D1 positive tumors were smaller than 2 cm (55.6%, p = 0.005), had a low incidence of lymph node metastasis (38.2%, p = 0.04) and were associated with increased DFS of >150 months (p = 0.04). Overexpression of c-myc (90%) was linked with a higher incidence of node negativity (58.3%, p = 0.006) and increased DFS (p = 0.04). p27 over expression was associated with decreased lymph node metastasis (p = 0.04). P21 and p57 positive tumors were related to decreased DFS (p = 0.04). Though p16 was overexpressed in 76.6%, this did not reach statistical significance with DFS (p = 0.06) or nodal status (p = 0.07).

**Conclusion:**

Aberrant cell cycle protein expression supports our view that these are important pathways involved in the etiopathogenesis of MBC. Tumors with overexpression of Cyclin D1 and c-myc had better outcomes, in contrast to tumors with overexpression of p21, p57, and PCNA with significantly worse outcomes. P27 appears to be a predictive marker for lymph nodal status. Such observation strongly suggests that dysregulation of cell cycle proteins may play a unique role in the initiation and progression of disease in male breast cancer. Such findings open up new avenues for the treatment of MBC as a suitable candidate for novel CDK-based anticancer therapies in the future.

## Introduction

Male breast cancer (MBC) remains a rare yet potentially fatal disease, accounting for less than 1% of mammary neoplasia [1-4] and 0.17% of all tumors in men [5], yet this number is rising [2,6,7]. While the incidences of MBC in North America and Western Europe remain low, the proportion of MBC cases is as high as 15% in sub-Saharan Africa [6]. The majority of the baseline knowledge and treatment protocols of male breast cancers are largely extrapolated from the treatment and behavior of female breast cancers (FBC) [1,7] as MBC behaves similarly to FBC in post-menopausal women [8]. The prevalence of MBC increases with age and the presentation occurs at an average age of 60 years, a decade later than in females. The majority of patients present with a painless, firm subareolar mass, tumors usually larger than 2 cm in diameter, and there may be fixation to skin. Pathologically, invasive ductal carcinoma (93.7%) is the predominant subtype, and lobular carcinoma is rare (1.8%) [5,9]. Nevertheless invasive ductal carcinoma of MBC is distinctly different from that in females in both presentation and immunophenotype [10]. Risk factors of MBC include testicular disease, benign breast conditions, age, Jewish ancestry, family history, liver disease, obesity, electromagnetic field radiation, infertility, and the strongest association being Klinefelters syndrome [7,11].

Due to the rarity of MBC, limited information is available [1,3]. Typically men with breast cancer have a longer duration of symptoms than women [12]. With a lack of awareness and the advanced stage at presentation, such delay in diagnosis often causes a worse prognosis than FBC [13]. Consequently MBC patients have a mortality of 5-10 years in 36-75% of cases [13]. Because of uninformed population, the need to implement means of communication to notify males and urge imaging studies is greatly important as a means to lower the risk of worse prognosis in MBC [5]. This risk is further amplified as men with breast cancer have a significantly higher risk for a secondary malignancy in comparison with the general population [13].

Studies in our laboratory confirm that male breast cancers display distinct immunophenotypic differences in comparison to female breast cancers [1]. The male breast cancers despite being high-grade neoplasms remain estrogen and progesterone receptor positive and cerbB2 and p53 negative [1]. Thus, it is postulated that alternative pathways of carcinogenesis are involved in the development and progression of male breast cancers [1]. Such pathways may implicate cell cycle dysregulation, apoptosis, growth factor pathway and/or androgen receptor pathway [1]. Deregulation of cell cycle control is central to our understanding of the development and progression of all human malignancies [14]. These proteins that play key roles [15-18] in the cell cycle regulation have therefore been the interest of our current study. We investigated the expression of CyclinD1, PCNA, c-myc, Ki67, p21, p27, p57, p16 and correlated the expression level of these factors with clinicopathological factors, such as lymph node status, tumor size, stage of the disease and disease free survival, as in many female breast cancer studies these four clinico-pathological parameters have proven to be of high prognostic value [16,18]. Additionally, we compared the outcomes of our study with results, found in the published literature about female breast cancer, to see if there are any major trends, which are unique to male breast cancer. The overall goal of this study was to fill major gaps in knowledge regarding the role of the cell cycle proteins in the etiopathogenesis of male breast cancer. This study is an extension of our established work on immunophenotypic characterization and angiogenesis in male breast cancer in Saskatchewan [1,10].

## Materials and methods

Seventy-five cases of primary male breast cancers were identified using the records of the Saskatchewan Cancer Agency over a period of 26 years (1970-1996). The clinicopathological profiles of these cases are identical to the previous published data from our laboratory [Additional file 1]. 59 of these cases had formalin fixed, paraffin embedded tissue blocks available for the purposes of this study. All cases were reviewed and graded according to Bloom-Richardson criteria for female breast cancers on a routine hematoxylin-eosin-stained slide.

**Figure 1:**
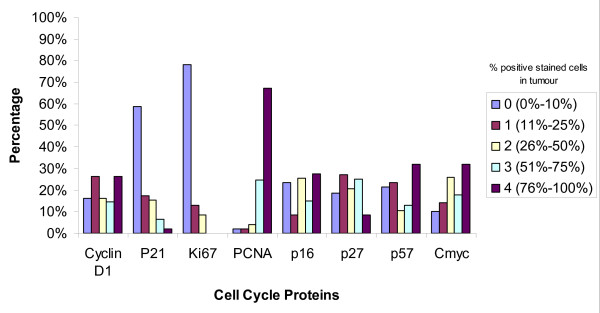
**Percentage of Cell cycle protein expression in the tumor cells**. X-axis displays: the expression of the cell cycle proteins CyclinD1, p21, Ki67, PCNA, p16, p27, p57, and c-myc. Y-axis displays: the percentage of positive stained cells in the tumor, where: 0 = no stain, up to 10% positive cells. 1 = light stain, 11-25% positive cells. 2 = moderate stain, 26-50% positive cells. 3 = heavy stain 51-75% positive cells. 4 = intense stain 76-100% positive cells.

Immunohistochemical studies were performed on a representative deparaffinized tissue section by the avidin-biotin-peroxidase (ABC) technique after antigen retrieval using appropriate positive and negative controls in all cases. Negative controls were obtained by omission of the primary antibody from the staining procedure. The antibodies used with their sources and dilutions are listed in Table 1. The immunohistochemical expression of PCNA, Ki67, p27, p16, p57, p21, cyclin-D1 and c-myc were analyzed on a semi-quantitative basis. As seen in figure 1, each slide was rated on a four-point scale: 0, no stain (up to 10% positive cells); 1, light (11-25% positive cells); 2, moderate (26-50% positive cells); 3, heavy (51-75% positive cells); 4, intense stain (76-100% positive cells). The cells were considered positive when more than 10% of them were stained with the respective antibodies.

**Table 1:** Antibodies examined in this study.

ANTIBODY	CLONE	DILUTION	SOURCE
PCNA	PC10	prediluted	Ventana
Ki67	MIB-1	1/100	Immunotech
p27	SX53G8	1/20	Dako
p16	F-12	1/75	Oncogene
p57	25B2	1/25	Novacastra
p21	EA10	1/5	Oncogene
cyclin-D1	P2D11F11	prediluted	Ventana
c-myc	9E11	1/200	Novacastra

This table lists the antibodies used in this study with clone, dilution ratio, and source.

**Figure 2:**
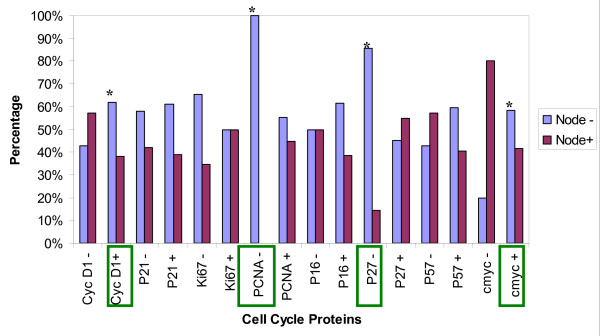
**Cell cycle protein expression in Node positive and Node negative tumors**. X-axis displays: the expression of positive and negative tumors for cell cycle proteins CyclinD1, p21, Ki67, PCNA, p16, p27, p57, and c-myc. Y-axis displays: the node negative and node positive tumors. Statistical significance * p < 0.05.

**Figure 3:**
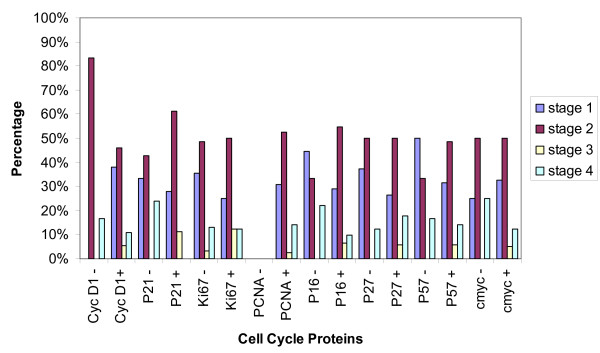
**Cell cycle protein expression and Stage of the disease**. X-axis displays: the expression of positive and negative tumors for cell cycle proteins CyclinD1, p21, Ki67, PCNA, p16, p27, p57, and cmyc. Y-axis displays: the four stages of disease. Statistical significance * p < 0.05.

**Figure 4:**
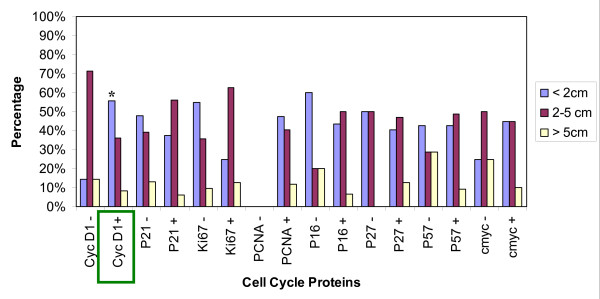
**Cell cycle protein expression and Tumor Size**. X-axis displays: the expression of positive and negative tumors for cell cycle proteins CyclinD1, p21, Ki67, PCNA, p16, p27, p57, and cmyc. Y-axis: the tumor size data includes: less than 2 cm, 2-5 cm, and more than 5 cm. Statistical significance * p < 0.05.

**Figure 5:**
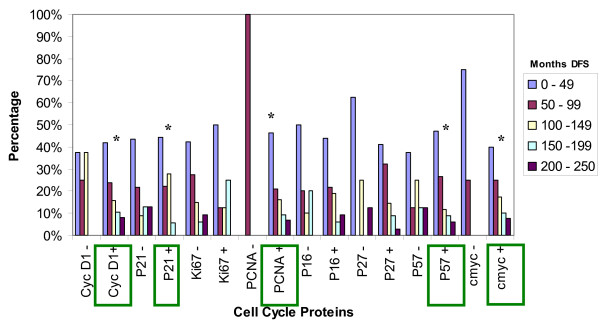
**Cell cycle protein expression and the Disease Free Survival (DFS, counted in months)**. X-axis displays: the expression of positive and negative tumors for cell cycle proteins CyclinD1, p21, Ki67, PCNA, p16, p27, p57, and cmyc. Y-axis displays: the duration of disease free survival (DFS, counted in months). Statistical significance * p < 0.05.

Statistical analysis using the Statistical Package for the Social Sciences (SPSS) version 16 compared the immunohistochemical expression of these proteins to the following prognostic clinico-biological parameters: a) nodal status (Figure 2), b) stage of the disease (Figure 3), c) of tumor size (Figure 4), and d) disease free survival (Figure 5). Disease free survival (DFS) was defined as the interval between primary treatment to the first recurrence or death. Statistical significance of the immunohistochemical scores were calculated using the Fisher's exact test. Statistical significance of the differences between the cases demonstrating positive and negative cell cycle protein expression in each of the clinicobiological parameter assessed was calculated using the two sample Student'st-test. A P-value of less than 0.05 was considered statistically significant.

This study was conducted with ethics approval from the University of Saskatchewan Advisory Committee on Human Experimentation.

## Results and discussion

Management protocols for male breast cancer patients have been modeled on traditional female breast cancer treatment regimes. However, it is becoming more apparent with increased work in this area that the male breast cancers do not seem to behave similar to female breast cancers. Study of male breast cancers in our own laboratory has revealed that despite the majority of these neoplasms being high-grade cancers, they retain the expression of estrogen and progesterone receptor antibodies and are also less likely to over express Erb-B2 and/or p53 in contrast to high grade female breast cancers. This therefore surmises that the current pathways of treatment protocols applicable in women that are directly linked to ER up-regulation leading to activation of downstream targets such as p53 and/or Erb-B2 does not hold validity in the case of male breast cancers. Thus, alternative pathways such as cell cycle dysregulation or androgen receptor alterations are perhaps involved in the development and evolution of male breast cancer. As deregulation of cell cycle control is central to our understanding of the development and progression of all human malignancies [14], this was explored in our laboratory in this study protocol.

### Cell Cycle

**Figure 6:**
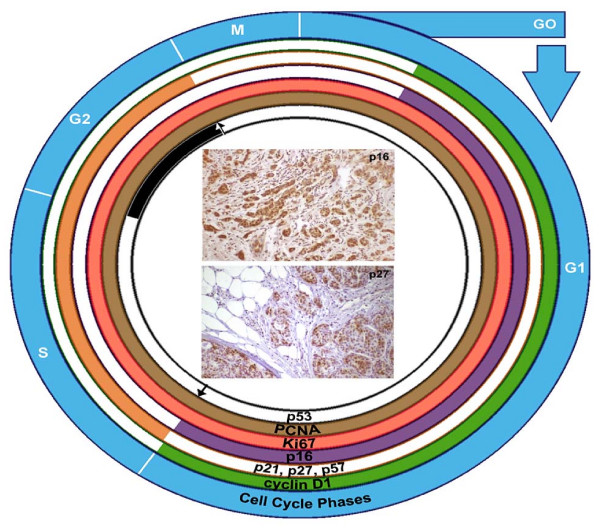
**Schematic illustration of the regulatory proteins in the cell cycle phases**. The cell cycle is illustrated (outer blue circle) schematically through its various phases--G_1_/S, G_2_/M. (G_1 _= growth phase 1; S = synthesis; G_2 _= growth phase 2; M = mitosis). The proteins studied are color coded to the most prominent phase of their action in the cell cycle: Cyclin D1 is green, G_1. _p21, p27, p57 are orange, S/G_2. _p16 is violet, G_1. _Ki67 (coral) and PCNA (brown) all phases. P53 is black, checkpoint G_2_/M. The insert are two photomicrographs of the immunohistochemical expression of p27 and p16 in the malignant breast cancer cells at a medium magnification ×150.

The cell cycle is a defined set of phases and checkpoints through which a proliferating cell must pass prior to division. As illustrated in figure 6 the four phases are *gap 1 (G*_1_), *synthesis (S)*, *mitosis (M) *and *gap 2 (G*_2_). In the G_1 _phase, the cell grows in preparation for DNA synthesis. It follows therefore that the subsequent phase, the S phase, is where DNA synthesis occurs. After this, the cell goes through the second gap phase, where the cell grows in preparation for its physical division. This division occurs in the M phase. After division, the daughter cells may continue proliferating by entering the G_1 _phase; alternatively, the cell may enter a fifth phase labeled G_0_. In G_0_, cells are quiescent (non-dividing); G_0_cells may experience cessation of proliferation temporarily or in permanence. There are three important checkpoints: *G*_1_, *G*_2_, and *metaphase*. At the G_1 _checkpoint, mechanisms verify that the cell has grown sufficiently and that the environment is suitable for DNA synthesis. At the G_2 _checkpoint, mechanisms verify that the DNA has successfully replicated, that the cell is big enough and that the environment is suitable for actual cell division. The metaphase checkpoint verifies that the chromosomes are aligned on the spindle during mitosis. If these conditions cannot be satisfied at their respective checkpoints, there is cessation of the cell cycle. Cells regulate growth through complex signaling pathways that act to maintain and integrate sequence of DNA replication (DNA synthesis, S-phase) that precedes mitosis in the cell cycle. Cyclin-dependent kinase enzymes (CDKs) determine cell cycle proliferation, such that their activation depends on an association with a phase specific protein. DNA damage activation of "checkpoints" ensure genomic integrity through inhibition of CDKs to effect a cell cycle arrest and repair prior to replication (G1 checkpoint), or mitosis (G2 checkpoint), with apoptosis constituting an alternative pathway of eliminating DNA damaged cells. Loss of "checkpoint functions" is a hallmark of many human cancers where there is replication and segregation of damaged DNA. P21 functions as a universal cyclin-dependent kinase inhibitory protein (CDK1) with an affinity for G1 and G2 cyclin-CDK complexes, thus acting as "checkpoint proteins" at the G1 and G2 levels (figure 6).

The molecular machinery which controls the cell cycle relies on a delicate balance between factors supporting growth and factors supporting stasis. The growth (or lack thereof) we observe by an individual cell is a reflection of the net sum of all growth promoting and inhibiting factors in its local environment. The factors of interest in this study are cyclin-D1, PCNA, Ki67, p16, p21, p27, p57 and c-myc.

### Cyclin-D1

Cyclins are responsible for controlling entry and progression through the cell cycle, specifically regulating the G1-S phase transition (figure 6). Induction of this cyclin shortens the G1 phase and consequently increases the number of cells passing through this checkpoint [19,20]. These proteins complex with (and thus activate) cyclin-dependant kinases (CDKs). Varying levels of different cyclins and CDKs are associated with progression through each of the important transitions in the cell cycle, and can be associated to tumor grade [21]. Cyclin D1 acts as one of the most commonly overexpressed oncogenes in breast cancer, found in 30-60% of primary ductal adenocarcinoma and universally overexpressed in lobular carcinomas [22,23]. The effects exerted by these diverse proteins include: altering activity of enzymes, altering affinity between proteins, altering affinity between protein and DNA, altering the metabolism of proteins. What effect a CDK may have depends not only on the protein itself, but also the environment, and the substrates involved [14,24-27].

Aberrant expression of cyclinD1 protein is a common feature in female breast cancers [24-27]. As a result of alternative splicing of the transcript CCND1, two isoforms of cyclin D1 exist: the conventional cyclin D1a and cyclin D1b [28]. In FBCs while high cyclin D1a levels are not associated with recurrence or metastases, high cyclin D1b levels are associated with poor survival and can predict disease outcomes. High cyclin D1a are found predominantly in ER-positive tumors and are inversely correlated with Ki67 with little impact on disease outcome. In contrast, elevated cyclin D1b expression was independently associated with adverse clinical outcomes including recurrence, distant metastasis and decreased survival thereby identifying a unique subset of tumors associated with increased disease progression [28].

In our study 83.7% of the cases were positive for cyclin-D1 overexpression (figure 1). Proven by several studies, female breast cancers also show high expression of Cyclin D1 [[14] (28%), [24](48.3% >5%), [25](59% >5%), [27,29] (65% >5%), [30] (66.7% >10%), [31,32]]. Cyclin D1 positive tumors seemed to be less likely associated with lymph node metastasis (38.2% vs. 57.1% in cyclin D1 negative tumors at p = 0.04, figure 2). This is in contrast to studies in female breast cancer that do not find a statistical significant correlation between cyclin D1 and metastatic disease and axillary lymph node involvement [19,25,29], yet there was an association between the expression of cyclin D1b and distant metastasis [28]. There was a strong tendency for cyclin D1 positive male breast tumors to be smaller than 2 cm [25,29] (55.6% vs. 14.3% of cyclin D1 negatives at p = 0.005, figure 4). Female breast cancers do not seem to have this correlation [25,29]. There was no correlation of CyclinD1 overexpression and the stage of the disease (figure 3). As seen in figure 5, none of the patients having a CyclinD1 negative tumor had a DFS over 150 months, in comparison to 18.4% in the CycD1 positive group (p = 0.04). In the published literature this is controversial and inconclusive. In some studies no correlation between CyclinD1 and DFS could be found[14,19,26] while Gillet et al has shown moderate/strong staining for CyclinD1 was associated with improved DFS and overall survival relative to tumors that stained weakly or negatively[32]. Yet negative Cyclin D1 tumors had an adverse prognosis with poor outcomes especially if they were ER negative tumors as well [33]. Our study shows that negative Cyclin D1 tumors are associated with adverse prognosis of increased incidence of lymph node metastasis, larger tumors, and decreased DFS.

### PCNA

Proliferating cell nuclear antigen (PCNA) is a protein which forms a ring around a portion of DNA serving to anchor various DNA replication and repair proteins and regulates proliferation throughout the cell cycle [34-36] (figure 6). PCNA expression was elevated in our study in 98% of the cases, with 67.4% showing intensive staining (figure 1). Due to the low percentage of PCNA negative cases, a comparison of the clinicopathological parameters between the positive and negative group was not feasible. This elevated expression of PCNA is also seen in studies on female breast cancers, where 71.4%-100% of cases were considered PCNA positive [17,37] Mean proliferating index was 76.1%, with a range from 0-100%. Compared to results in female breast cancer, this value seems to be far above the range of results reported by others (10.2% -28.7%) [34]. In the female breast cancer literature the correlation between the range of proliferating indexes [PI] and classical prognostic factors such as tumor size and nodal status is controversial. Some authors found strong, statistically significant correlation between PI, PCNA or Ki67 level and tumor size or nodal status [34]. The majority of investigators however, think that such relationship do not exist [34]. In our study in male breast cancer the PCNA was positive in 55.3% of node negative tumors and 44.7% of node positive tumors, p = 0.0001(figure 2). As far as size of the tumor was concerned no significant statistical significance could be found with 47.6% positive PCNA expression in tumors with a size less than 2 cm (figure 4). However, PCNA overexpression was associated with decreased DFS (98%, p = 0.004) indicating perhaps disease progression with increased adverse clinical outcome.

### C-myc

The c-myc gene is amplified and/or overexpressed in different frequencies in most human malignancy [38], though amplification occurs more frequently in metastases than in primary tumors [39]. A regulator of a cell's size and participant in cellular functioning such as growth, differentiation, apoptosis, and metabolism [38], this oncogene can both activate and repress specific genes [21]. The c-myc protein binds to DNA and activates transcription for many growth related genes (including CDKs). The myc protein is induced when a cell is stimulated to pass from the quiescent G_0 _state to the active G_1 _state. The normal myc gene is a protooncogene, thus, when it becomes dysregulated (mutation or other) it promotes uncontrollable cell division [40]. In our study of male breast cancer, c-myc was expressed in 90% of the cases (figure 1). Several critical issues regarding the significance of c-myc in human breast cancer still remain obscure. The frequencies of the expression levels vary greatly from one report to another (50-100%) [40]. In our study the percentage of cases being node negative seemed to be lower in c-myc negative cancers (20% vs. 58.3%, p = 0.006) in c-myc positives) as demonstrated in figure 2.

In a female breast cancer study on node negative tumors done by Schlotter, c-myc amplification appears to represent a prognostic marker to predict early recurrence [41]. Pich et al has reported a 107 month survival for c-myc negative cases and 52 months for c-myc positive male breast cancer patients [42]. As seen in figure 5, in our study all c-myc negative tumors had a DFS lower than 100 months, with only one living longer than 50 months(p = 0.04). There was no statistically significant association between c-myc protein levels and stage of the disease as seen in figure 3. Though not statistically significant, p = 0.08, 55% of tumors >2 cms were c-myc positive in our study (figure 4). Interestingly, Aulmann et al, 2002 using FISH and focusing on DCIS, detected amplification of c-myc in only 20% of the cases, but found a correlation of c- myc with increased tumor size and proliferation [40]. Further, In FBCs, high C-myc expression levels are correlated on one hand with larger sizes tumors but on the other hand with better survival [38]. Similar parallel trends are seen in our study, wherein c-myc overexpression though associated with larger tumor size, had lower incidence of lymph node metastasis and better DFS indicating a favorable prognosis.

### Ki-67

Ki67 nuclear antigen is associated with cell proliferation and is found throughout the cell cycle except the Go phase [16,35,43](figure 6) and has become recognized as a proliferation marker in breast cancer [44] where a higher percentage correlates with an increase in tumor grade[45]. In our study Ki67 expression was mostly negative (78.3%, figure 1). This is in contrast to high grade FBC with high Ki67 expression in 95% of the cases [37]. Within the numbers of tumors considered positive for Ki67 expression, the proliferating index (PI) ranged from 0%-40%, leading to a mean PI of 6.6%. The mean PI so falls within the range of values reported by others studying female breast cancers (6%-22%) [34,35]. In our study Ki67 negative cases had a higher tendency of being node negative (65.5% vs. 50% in positive cases, figure 2). Furthermore there was a trend of Ki67 negative tumors, having a size less than 2 cm with 54.8% of the Ki67 negative tumors being smaller than 2 cm, whereas only 25% of the Ki67 positives were of similar size. Though not statistically significant, in our study Ki67 positive tumors seemed to be associated with larger tumor size as seen in figure 4. This finding is congruent with literature stating 20-40% of MBC cases are positive for Ki-67 and when combine with androgen receptor negativity tend towards worse prognosis [45]. In female breast cancers, some authors found strong, statistically significant correlation between PI, PCNA or Ki67 level and tumor size as well as nodal status [34]. As already mentioned the majority of investigators, think that such relationship does not exist [16,36,37,43]. In this study the mean PI node negative male breast tumors was 2.73% (range 0%-10%) and 7.7% (range 0%-30%) in node positives (figure 2). Tumors with a size less than 2 cm had an IP of 3.95% (range 40%-100%) and those larger than 2 cm one of 9.3% (range 0%-40%). Remarkable is also the inverse correlation between Ki67 and PCNA within the tumors of this study. Also in studies about female breast cancer some authors have found a similar lack of correlation between the two indices [35,36]. Yet there are also studies that report the opposite. No significant correlations were observed between the Ki67 expression levels and tumor stage (figure 3) and DFS as demonstrated in figure 5. In our study, Ki67 does not appear to play a dominant role in disease progression or survival in male breast cancer.

### p21, p27, p57, p16

These proteins are part of the CDKN1A family; a family of proteins which broadly inhibits the activity of CDKs. As illustrated in figure 6 these proteins act as a brake for cell proliferation; their expression contributing to a cessation in the cell cycle, especially during the S and G_2 _phases [15,18,33,46,47].

p21 remained negative in 58.7% of our male breast cancer cases(figure 1). As other studies showed, there was no significant difference in this point concerning the female counterpart [46]. In our study 94.4% of tumors showing a p21 expression had a DFS shorter than 150 months (vs. 74% in the negative group, p = 0.04) as seen in figure 5. This trend of p21 negativity combined with a longer disease free survival in our cases of male breast cancer has also seen in a study of female breast cancer [48]. In another existing study the immunohistochemical expression of p21 was analyzed and compared between 27 cases of primary male breast cancer (MBC) and 101 cases of female breast cancer (FBC). A statistically significant difference in the immunostaining of p21 in male patients compared with females was found. Expression of p21Waf1 was observed in 19 of the 27 primary MBC (70.3%) vs. 29 of 101 FBC (29%) [47]. André et al has demonstrated the occurrence of p21-positive is significantly higher in MBC than FBC (FBC: 58% positive 42% negative vs. MBC: 4% negative 96% positive) [49]. This further strengthens the view that MBC and FBC probably have distinct tumor oncogenesis. The exact biological role of p21 expression remains unclear as there is no evidence of strong correlation with other cell cycle regulatory proteins or Ki-67 [49]. Yet, overall p21 positivity is associated with adverse outcomes as it is associated with decreased DFS.

p27 is a CDK inhibitor required for entry to S-phase. Loss of p27 is believed to contribute to oncogenesis [47]. Most often associated with cell cycle arrest [47,50], p27 maintains CDKs in an inactive state and thus blocks entry to the S-phase [51] and in tumors with a high estrogen receptor expression and low S-phase fraction has a high expression [47]. The level of p27 is not stagnant: the level rises as the cells exit the cell, proteolysis causes the levels to drop, and can be inactivated by cyclin sequestering [50]. P27 overexpresion in MBC could reflect a failing feedback attempt of a normal protein rather than be the result of an alteration in the p21 gene. Expression of p27 was noted in 81.2% of our cases (figure 1). A study about the role of p27 in human breast cancer cell lines showed that 5 out of 12 (41.7%) of the tested cell lines showed high level p27 expression [15]. Similar to our findings in Curigliano's study the immunohistochemical expression of p27 was analyzed and compared between 27 cases of primary male breast cancer (MBC) and 101 cases of female breast cancer (FBC). P27 immunoreactivity was detected in 26 of 27 male breast patients (96.2%) vs. 39 of 101 FBC (39.3%) [47]. A salient finding in our study is the high percentage (85.7%) of node negative cancers in the p27 negative group in comparison to 45.2% at p = 0.04 (figure 2). P27 expression in node negative cases suggests that p27 may be a predictive marker for lymph nodal status.

Expression of p57 was noted in 78.7% of our MBC cases (figure 1). P57 positives tumors showed a slightly higher tendency to be associated with node positivity (57.1% vs. 40.6% of the p57 negative cases, figure 2). As for tumor size (figure 4), 28.6% of the p57 negatives were larger than 5 cm in comparison to 9.1% in the p57 positive cases suggesting loss of p57 expression being associated with larger tumor size. Tumors that were p57 and p21 positive were associated with decreased DFS (p = 0.04) (Figure 5) indicating adverse outcomes.

### P16

p16 belongs to the CDKN2A family of proteins, another family of CDK inhibitors [18,52]. This family of proteins particularly inhibits the activity of active CDK4 and CDK6 [52]; thus, their inhibitory activity occurs primarily in the G_1 _phase where it accumulates and inhibits progression to the S-phase [53] (figure 6). Reduced expression of this protein is caused by its inactivation by deletion, mutation, or methylation [53]. Mechanisms leading to p16 overexpression is however not well understood. As p16 is inactivated in 85% of tumor-derived cells lines, it is classified as a tumour suppressor and is the second most common genetic mutation found in breast cancer [50]. In our study 76.6% of all tumors showed p16 expression (figure 1). Whereas this finding is not discrepant with another study about the expression of p16 in female breast carcinomas [52], there are other studies where half of the breast cancers in women failed to express p16 [18,54,55]. Neither the presence of p16 positive nuclei nor the lack of detectable staining in these studies was statistically significant with tumor size (figure 4), tumor differentiation or nodal status (figure 2). Though the higher percentage of large tumors >5 cms were associated with loss of p16 expression (20% vs. 6.7% in the positives) we could not find any statistical correlation between the expression of p16 and the DFS (p = 0.06) (figure 5), nodal status (p = 0.07) (figure 2) or the stage of the disease as demonstrated in figure 3. Thus similar to other investigators despite aberrant expression, the significance of the expression of P16 in male breast cancer could not be established [52,53,55].

## Conclusion

Male breast cancer is a rare yet potentially aggressive disease with a distinctive immunophenotype with alternative pathway for tumor oncogenesis distinct from female breast cancer. The management of MBC therefore necessitates different treatment regimes rather than the traditional FBC approaches. Our study confirms aberrant expression of cell cycle proteins in male breast cancers. Tumor cells with overexpression of Cyclin D1 and c-myc are associated with favorable outcomes while overexpression of p21, p57, and PCNA are linked with adverse outcomes. P27 appears to be a predictive marker for lymph nodal status. The exact role of p16 expression remains undetermined. These cell cycle protein markers may identify a unique subset of tumors that may be associated with aggressive disease. Dysregulation of cell cycle proteins may play a unique role in the initiation and progression of disease in male breast cancer. This opens up a new perspective for the treatment of MBC as a suitable candidate for novel CDK-based anticancer therapies in the future.

## Competing interests

The authors declare that they have no competing interests.

## Authors' contributions

All authors participated in the writing of this manuscript. RK is the corresponding and first author who was involved in the design and implementation of the study with overseeing of the data gathering and data interpretation. IF and TR are international exchange medical students who worked on this project during the summer of their elective here in our laboratory. JLS is an undergraduate summer student who was involved with extensive revisions and writing of this manuscript. SCK is the senior author of this paper.

## Supplementary Material

Additional file 1 **Appendix 1**. Table from Archives of Pathology & Laboratory Medicine: Vol. 127, No. 1, pp. 36-41. Click here for file
