# Supplementary figures and images for: Expression of cell cycle proteins in male breast carcinoma
Source: World J Surg Oncol. 2010 Feb 12;8:10. doi: 10.1186/1477-7819-8-10 (PMC2829567; doi:10.1186/1477-7819-8-10)

APPENDIX 1


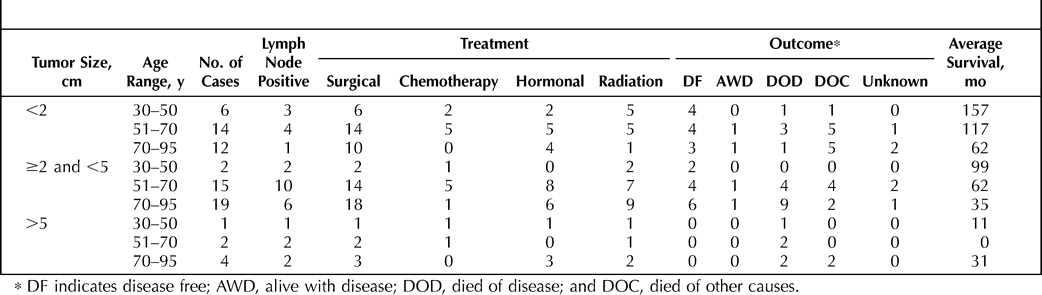


***Archives of Pathology & Laboratory Medicine: Vol. 127, No. 1, pp. 36-41***

Supplement: Additional file 1 — Appendix 1. Table from Archives of Pathology & Laboratory Medicine: Vol. 127, No. 1, pp. 36-41. [file 1477-7819-8-10-S1.DOC]
